# Lysine deacetylase inhibitors have low selectivity in cells and exhibit predominantly off‐target effects

**DOI:** 10.1002/2211-5463.13896

**Published:** 2024-10-31

**Authors:** Kiara E. Bornes, Marcus A. Moody, Thomas M. Huckaba, Megan C. Benz, Emily C. McConnell, Maryam Foroozesh, Van H. Barnes, Bridgette M. Collins‐Burow, Matthew E. Burow, Terry J. Watt, Tasha B. Toro

**Affiliations:** ^1^ Department of Chemistry Xavier University of Louisiana New Orleans LA USA; ^2^ Tulane University School of Medicine New Orleans LA USA; ^3^ Department of Biology Xavier University of Louisiana New Orleans LA USA

**Keywords:** HDAC6, HDAC8, HDACi, immunofluorescence, RNA‐seq

## Abstract

Lysine deacetylases (KDACs or HDACs) are metal‐dependent enzymes that regulate lysine acetylation, a post‐translational modification that is present on thousands of human proteins, essential for many cellular processes, and often misregulated in diseases. The selective inhibition of KDACs would allow for understanding of the biological roles of individual KDACs and therapeutic targeting of individual enzymes. Recent studies have suggested that purportedly specific KDAC inhibitors have significant off‐target binding, but the biological consequences of off‐target binding were not evaluated. We compared the effects of treatment with two of the reportedly most KDAC‐selective inhibitors, Tubastatin A and PCI‐34051, in HT1080 cells in which the endogenous KDAC6 or KDAC8 gene has been mutated to inactivate enzyme catalysis while retaining enzyme expression. Genetic inactivation results in much stronger deacetylation defects on known targets compared to inhibitor treatment. Gene expression analysis revealed that both inhibitors have extensive and extensively overlapping off‐target effects in cells, even at low inhibitor doses. Furthermore, Tubastatin A treatment led to increased histone acetylation, while inactivation of KDAC6 or KDAC8 did not. Genetic inactivation of KDAC6, but not KDAC8, impaired tumor formation in a xenograft model system, in contrast to previous reports with KDAC inhibitors suggesting the reverse. We conclude that the majority of observed biological effects of treatment with KDAC inhibitors are due to off‐target effects rather than the intended KDAC inhibition. Developing a truly specific KDAC6 inhibitor could be a promising therapeutic avenue, but it is imperative to develop new inhibitors that selectively mimic genetic inactivation of individual KDACs.

## Graphical abstract

Lysine deacetylases (KDACs or HDACs) regulate lysine acetylation. Genetic inactivation results in much stronger deacetylation defects on known targets compared to inhibitor treatment. Inhibitors have predominantly off‐target effects in cells, even at low inhibitor doses. The majority of observed biological effects of treatment with KDAC inhibitors are due to off‐target effects rather than the intended KDAC inhibition.

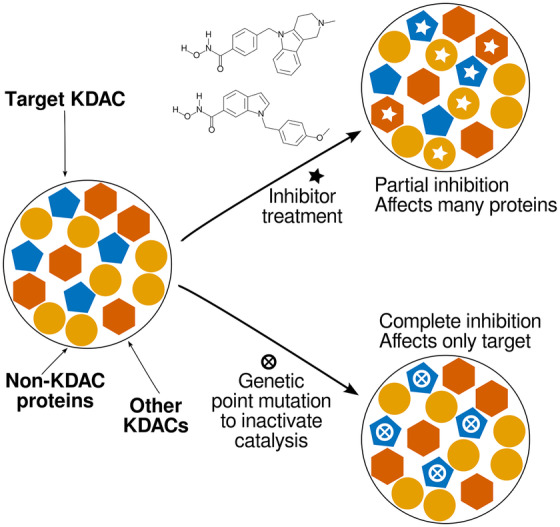

**Abbreviations**
GO: gene ontology
HDAC: histone deacetylase
KDAC: lysine deacetylase
PCI: PCI‐34051
Tuba: Tubastatin A
WT: wild‐type

Lysine acetylation is a reversible, highly regulated post‐translational modification that affects thousands of human proteins and is an important regulator of multiple cellular processes [1]. Lysine deacetylases (KDACs), also known as histone deacetylases (HDACs), are a family of 11 metal‐dependent enzymes in humans that are responsible for reversing lysine acetylation for normal cellular function [2]. Perturbations of this process ultimately lead to many aberrant phenotypes. KDAC6 (Uniprot KB: Q9UBN7) and KDAC8 (Uniprot KB: Q9BY41), in particular, have been linked to several disease states, including cancer [3, 4, 5].

Much of our knowledge concerning how individual KDACs function in cells and which target proteins are deacetylated by specific KDACs heavily relies on inhibitor‐driven experiments, which assume that inhibitors are selective in cells [6]. In addition to their importance for understanding KDAC function, KDAC inhibitors have been extensively developed and investigated as potential therapeutics. Despite the plethora of selective KDAC inhibitors reported in the literature and a well‐established link between KDAC misregulation and disease states, there has been limited success in demonstrating efficacy of KDAC inhibitors as a primary therapeutic strategy [4, 7, 8, 9]. Although a few pan‐KDAC inhibitors have been approved as drugs for cancer therapy, their success in the clinic has been limited and mostly relegated to combination therapy regimes, due to significant side effects and limited clinical benefit [10]. It is currently unclear whether KDACs are poor drug targets for mitigating diseases such as cancer, or whether the current suite of inhibitors are not adequate as therapeutics. Several lines of recently reported work suggest that the low success rate is potentially a systemic problem with KDAC inhibitors due to much lower biological specificity than indicated by *in vitro* assays [11, 12, 13, 14, 15, 16]. Furthermore, two drug candidates targeting KDAC6 and undergoing clinical trials were recently reported to exert their biological effects primarily via off‐target mechanisms, as were several drug candidates for other proteins [17].

Many small molecule KDAC inhibitors have been developed and touted as selective inhibitors based on comparing *in vitro* IC_50_ values among members of the KDAC family. These inhibitors have been used extensively in research to understand the roles of specific KDACs in cellular functions [5, 7, 18]. Despite the apparent potency and selectivity of these molecules *in vitro*, recent work suggests that they likely engage in off‐target binding both among other members of the KDAC family as well as other protein families. One report demonstrated that many traditional KDAC inhibitors can bind other metalloproteins present in cells and likely elicit off‐target effects, even at concentrations below those necessary for KDAC inhibition [11]. Other evidence suggests that KDAC6‐selective inhibitors can significantly inhibit histone‐modifying KDACs as well as specific unrelated proteins at doses commonly utilized in research reports, with possible implications for cancer models [12, 15, 16]. Another recent report demonstrated that similar inhibitors can significantly alter free metal concentrations in cells, potentially leading to far‐reaching consequences for cell function [13]. However, these studies focused on establishing specific off‐target binding of the inhibitors and did not generally determine what, if any, biological consequences result from off‐target binding.

Here, we focus on Tubastatin A and PCI‐34051, widely used competitive inhibitors with reported selectivity for KDAC6 and KDAC8, respectively (Table 1). Tubastatin A has a reported *in vitro* IC_50_ value of 15 nM for KDAC6, which is >50‐fold lower than any other family member [19]. PCI‐34051 has a reported *in vitro* IC_50_ value of 10 nm for KDAC8, which is almost 300× lower than the value for KDAC6 [20]. An important caveat for interpretation of these IC_50_ values is that most KDACs typically function in large complexes that can alter their *in vivo* activity in ways not reflected when measuring *in vitro* inhibitor binding [2]. The initial characterization of these inhibitors did not include information about selectivity outside of the KDAC family [19, 20]. In the study systematically evaluating off‐target binding, PCI‐34051 was selective for KDAC8 with no measurable off‐target binding [11]. Of the tested KDAC6 inhibitors, Tubastatin A had among the least binding to non‐KDACs but demonstrated significant interaction with KDAC10, which would not have been predicted from the initial *in vitro* IC_50_ measurements [11, 19].

**Table 1:** Inhibitor data and structures. n.d., not determined.

Compound	Reported *in vitro* IC_50_ (μm) for KDAC											Structure
	1	2	3	4	5	6	7	8	9	10	11	
Tubastatin A [19]	16	>30	>30	>30	>30	0.015	>30	0.85	>30	>30	>30	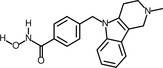
PCI‐34051 [20]	4	>50	>50	n.d.	n.d.	2.9	n.d.	0.01	n.d.	13	n.d.	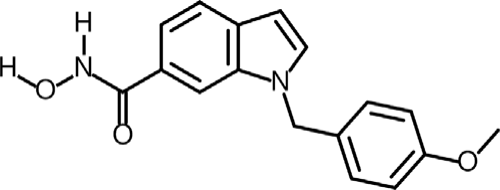

We previously generated HT1080‐derived cell lines in which the catalytic activity of specific KDACs have been inactivated by introducing missense mutations in the endogenous genes corresponding to these proteins [14]. The resulting cell lines retain approximately endogenous expression of the targeted KDAC and the ability to perform any functions of the KDAC except catalytic hydrolysis of acetyllysine. The 8CDm line (HT1080 KDAC8H143A) expresses catalytically inactive KDAC8. In the 6CD2m line (HT1080 KDAC6H611A), the second catalytic domain of KDAC6 has been inactivated, as this domain has been linked to most of the known activity of KDAC6, including tubulin deacetylation [14, 21]. These cell lines avoid the potential for off‐target effects that can occur in inhibitor studies. Initial characterization of these cell lines demonstrated the expected increase in acetylation of known substrates. Surprisingly, treatment of wild‐type (WT) HT1080 cells with either Tubastatin A or PCI‐34051 revealed that changes in gene expression, and the associated functions from gene ontology analysis, are dramatically different from the response to genetic inactivation of the targeted KDAC [14]. An implicit hypothesis in the KDAC field is that inhibitors targeting KDACs will not interact with other protein families, and therefore, selectivity only needs to be evaluated against other KDACs. The observations of interactions with other metalloproteins and extensive gene expression changes instead suggest that these KDAC inhibitors are not truly selective in cells. In this report, we have characterized cellular consequences of genetic inactivation of KDAC6 or KDAC8 compared to treatment of cells with the KDAC‐selective inhibitors Tubastatin A or PCI‐34051. Our results indicate that the assumptions made about the selectivity of frequently utilized KDAC inhibitors are invalid, as Tubastatin A and PCI‐34051 demonstrate predominantly off‐target effects in cells that have important consequences for cellular function, with major implications for our understanding of the roles of KDACs in cell biology and disease.

## Materials and methods

### Cell culture and inhibitor treatment

WT HT1080 cells (ATCC, Manassas, VA USA) and the derived cell lines, 6CD2m (HT1080 KDAC6H611A) and 8CDm (HT1080 KDAC8H143A), were cultured as previously described [14]. Cells were grown in Modified Essential Medium Eagle (MEM; Corning, Corning, NY USA) containing 10% fetal bovine serum (Cytiva, Marlborough, MA USA) under 5% CO_2_. Where indicated, cells were treated with Tubastatin A (Cayman Chemical, Ann Arbor, MI USA) or PCI‐34051 (Cayman Chemical) in cell culture for 48 h or 14 days, as noted. Concentrations are reported in the relevant figure captions. For longer treatment, inhibitor was replenished each time the cell were passaged or media was changed, at least every 3 days.

### Immunoblotting

All immunoblots were performed as previously described [14]. Approximately 25 μg lysate was loaded for each sample. The following primary antibodies (from Cell Signaling Technologies, Danvers, MA USA, unless otherwise noted) were used in this study: SMC3 (5696), SMC3‐acetyl‐K105/K106 (Sigma Aldrich MABE1073, St. Louis, MO, USA), α‐Tubulin (3873), α‐Tubulin‐acetyl‐K40 (5335), Histone H3 (4499), Histone H3‐acetyl‐K9 (9649), Histone H3‐acetyl‐K18 (13998), Histone H3‐acetyl‐K27 (8173), Histone H2B (12364), and Histone H2B‐acetyl‐K5 (12799). All antibodies were used at a dilution of 1:1000, except Histone H3, which was used at a dilution of 1:2000. Immunoblot images were processed uniformly with a linear intensity range and for the maximum intensity range without exceeding saturation in any bands, except in long exposures in which the intensity range of the faintest non‐zero band in the short exposure was maximized while overexposing the more intense bands.

### Immunofluorescence

Cells were grown on plasma‐treated glass coverslips using normal growth conditions until 30–50% confluent. Cells were fixed by diluting formaldehyde to 4% directly in growth media and incubating for 15 min at room temperature. Coverslips were then washed with PBS and incubated in ice‐cold methanol for 10 min. Cells were washed again and blocked with 5% goat serum in PBS for 30 min at room temperature. Cells were incubated with antibodies against α‐Tubulin (1:50) and α‐Tubulin‐acetyl‐K40 (1:100) in PBS containing 5% goat serum for 2 h at room temperature. Cells were washed in PBS and incubated with AlexaFluor α‐mouse‐488 and α‐rabbit‐568 (Thermo Fisher, Waltham, MA USA) in PBS containing 5% goat serum for 1 h at room temperature. After washing in PBS, coverslips were mounted using SlowFade™ diamond antifade mountant with DAPI (ThermoFisher). Cells were visualized using a Nikon A1 microscope. Fluorescence excitation occurred using an EXFO LED array at 50% intensity and filtered through Nikon filter cubes appropriate for the specific fluorophores. Images were acquired through a Nikon PlanAPO 60× (1.40 NA) oil objective via an Andor EMCCD camera. All imaging was carried out with 100 ms exposure time for each channel and the gain set to zero on the EMCCD camera. Paired raw images were opened as a stack in ImageJ, a freehand line was drawn around the perimeter of each cell, and the mean grayscale value inside each cell was measured by the software for both channels. Distributions were compared by multiple *t*‐tests with Bonferroni correction.

### Gene expression analysis

RNA‐seq analysis was performed as previously described, except that a false discovery rate adjustment of *α* = 0.001 was used in this study [14]. Briefly, 3–4 biological replicates of at least 3.5 μg total RNA were rRNA‐depleted, used to synthesize cDNA, sequenced to a minimum of 4 × 10^7^ reads per sample, and the resulting data processed as previously described [14].

### Gene ontology (GO) analysis

The basic ontology set 2022‐10‐07 was utilized with the *is_a* and *part_of* relations within the *biological_process* group of the GO human annotation set 2022‐09‐17. The reference set was all possible human genes as annotated for RNA‐seq analysis. Statistically significant up‐regulated and down‐regulated genes compared to untreated wild‐type cells were analyzed independently for each cell line and treatment. A one‐tailed Fisher Exact test for overrepresentation of genes within each GO term was calculated, using an initial significance threshold of *P* = 0.01 in SciPy 1.12 [22]. Subsequently, we utilized the elim method with *P* = 0.01 with the Bonferroni correction to identify the most specific GO terms associated with the expression changes [23]. GO graphs were prepared using Graphviz [24].

### Xenograft studies

Female SCID/beige immunocompromised mice (6–8 weeks old) were procured from Charles River Laboratories (Wilmington, MA). HT1080 WT, 6CD2m, or 8CDm cells were harvested from tissue culture and subsequently washed with PBS/EDTA solution, resuspended in 250 μL of PBS and mixed with 250 μL of Vitrogel (The Well Bioscience Inc, North Brunswick Township, NJ, USA). Subcutaneous injections of 2.5 × 10^6^ cells per injection were administered bilaterally into the flanks of five mice per cell line. To ensure the well‐being of the animals and to minimize any potential discomfort, all procedures involving the mice were conducted under anesthesia using a mixture of isoflurane and oxygen. Following the injections, mice were monitored daily for the formation of palpable tumors. Tumor volume was measured using a caliper and calculated as 1/6 * π * length * width * depth. Distributions of tumor volumes were compared to wild‐type using a t‐test with a threshold of *P* < 0.01 with Bonferroni correction. On day 21 post cell injections, the mice were humanely euthanized following ethical guidelines, using cervical dislocation following CO_2_ exposure. The second experiment was performed similarly, except cells were mixed with Cultrex (Biotechne, Minneapolis, MN, USA) and injected into mammary fad pads. Mice were euthanized, by CO_2_ exposure prior to cervical dislocation, when tumors became too large or ulcerated, mice became ill, or no tumor developed within 130 days. Growth rates were determined by the slope of a linear fit to log_2_ of data from initial tumor formation until the tumor exceed 256 mm^3^. Significance was determined by *t*‐test. Ethical approval for this study was obtained from the Tulane University Institutional Animal Care and Use Committee (reference number 6566). All procedures involving animals were conducted in compliance with Louisiana and Federal laws, standards of the US Department of Health and Human Services, and guidelines established by Tulane University Animal Care and Use Committee.

## Results

### KDAC‐selective inhibitors incompletely inhibit the target KDAC

**Fig. 1:**
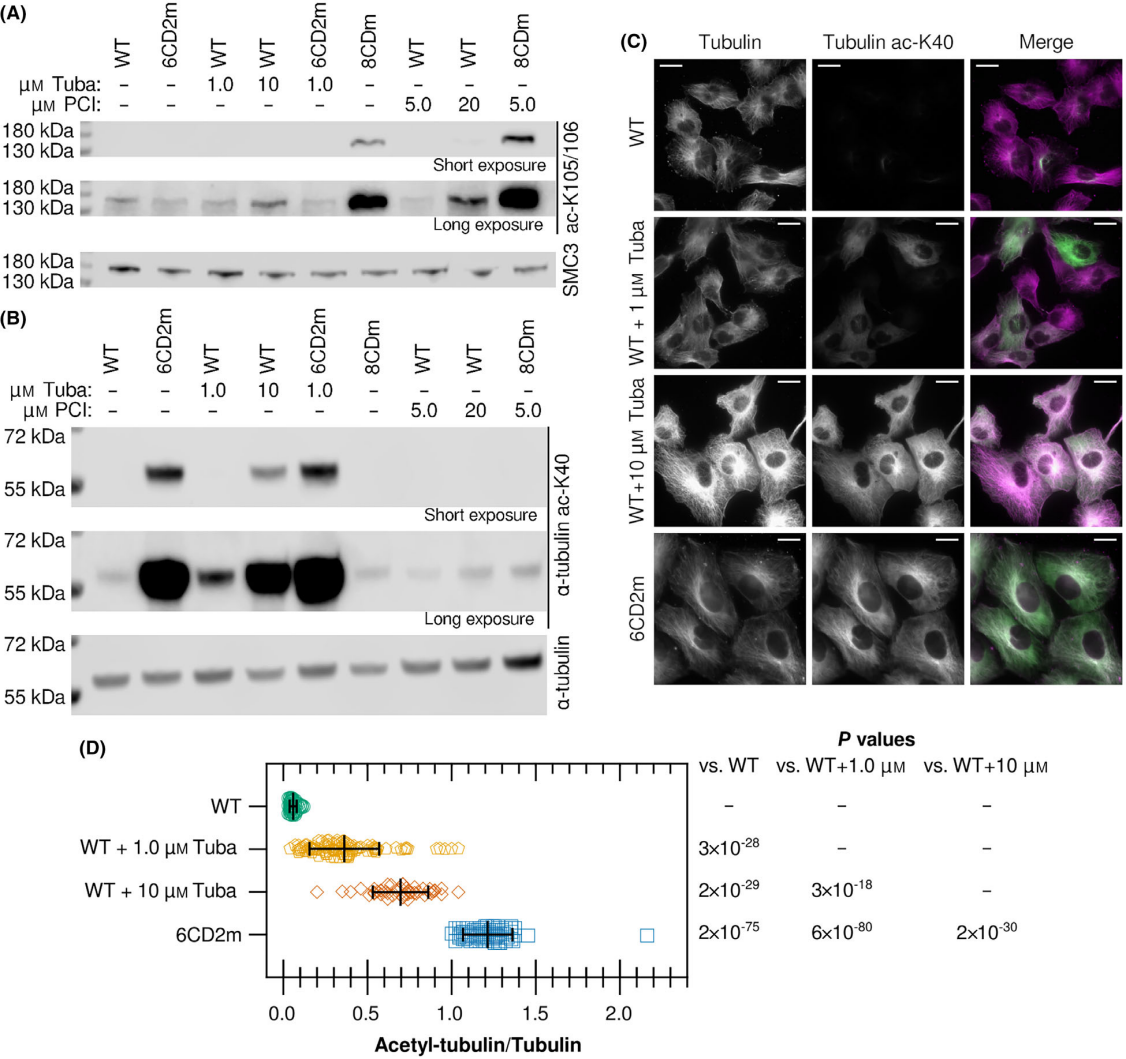
KDAC6 and KDAC8 activity persists despite treatment with selective KDAC inhibitors. Cell lines were treated with either Tubastatin A (Tuba) or PCI‐34051 (PCI) for 2 days to compare acetylation levels of previously characterized substrates. (A) Representative immunoblots of lysates showing levels of SMC3 K105/K106 acetylation for each condition. Total SMC3 signal served as a loading control. Top and middle panels are different exposure levels of the same blot to allow for comparison of both weak and strong bands. (B) Representative immunoblots of lysates showing levels of α‐tubulin K40 acetylation for each condition. Total α‐tubulin signal served as a loading control. Top and middle panels are different exposure levels of the same blot to allow for comparison of both weak and strong bands. (C) Representative images of immunofluorescence of fixed cells using antibodies against α‐tubulin (magenta in merge) and α‐tubulin acetylated at K40 (green in merge). Gray images of each channel are normalized to the same maximum intensity. The intensity range of each channel was individually maximized for merged images, to more effectively show relative localization (white). Scale bars represent 20 μm. (D) Signals from immunofluorescence images were quantified for both α‐tubulin and acetyl‐K40 α‐tubulin. The ratio of acetyl‐tubulin/tubulin was calculated for individual cells (*n* ≥ 45 for each group). Whiskers represent the mean and standard deviation. Significance was evaluated by multiple *t*‐tests with Bonferroni correction.

To compare genetic inactivation and chemical inhibition of KDAC8, the known KDAC8 substrate SMC3 was used [6, 25, 26]. We compared the acetylation level of SMC3 K105/K106 in 8CDm cells (with genetically inactivated KDAC8) to WT cells treated with either 5.0 μm or 20 μm PCI‐34051, consistent with the dosing range used in the initial characterization of the molecule [20]. Genetic inactivation of KDAC8 resulted in much greater accumulation of acetylated SMC3 than did inhibitor treatment, even when a high concentration of PCI‐34051 was used (Fig. 1A). Notably, we detected a slight increase in acetyl‐SMC3 upon the higher dose of Tubastatin A treatment compared to WT; however, there was not an increase in the 6CD2m cell line.

Similarly, we compared the effect of genetic inactivation of KDAC6 to treatment with Tubastatin A on the accumulation of acetyl‐tubulin, the most well‐characterized substrate of KDAC6 [27, 28]. We compared acetylation levels of α‐tubulin K40 from lysates of the 6CD2m cell line (expressing inactive KDAC6 second catalytic domain) to WT cells treated with either 1.0 μm or 10 μm Tubastatin A, consistent with initial reports characterizing Tubastatin A [19]. Treatment with 1.0 μm Tubastatin A resulted in only a slight increase in acetyl‐tubulin. Increasing the dose to 10 μm led to a larger accumulation of acetyl‐tubulin, but still much less than observed in 6CD2m cells, suggesting that Tubastatin A only partially inhibits KDAC6 in cells (Fig. 1B). As expected, neither genetic inactivation of KDAC8 (8CDm) nor PCI‐34051 treatment resulted in an increase in acetyl‐tubulin.

This trend is also apparent by immunofluorescence, as the ratio of acetylated α‐tubulin to total α‐tubulin in microtubules is much higher in 6CD2m cells than in WT cells treated with Tubastatin A (Fig. 1C,D). Notably, the distribution of acetyl‐tubulin/tubulin signal was much wider in the Tubastatin A‐treated cells compared to the WT or 6CD2m cell line, demonstrating that there is considerable cell‐to‐cell variability in KDAC6‐inactivation in response to drug treatment in cells even after 2 days of treatment. Upon genetic inactivation of KDAC6, virtually all microtubules appear to be acetylated, in contrast to only a subset of cells having predominantly acetylated microtubules, even at the high Tubastatin A dose (Fig. 1C).

### KDAC‐selective inhibitors do not recapitulate the gene expression patterns of genetic KDAC inactivation

**Fig. 2:**
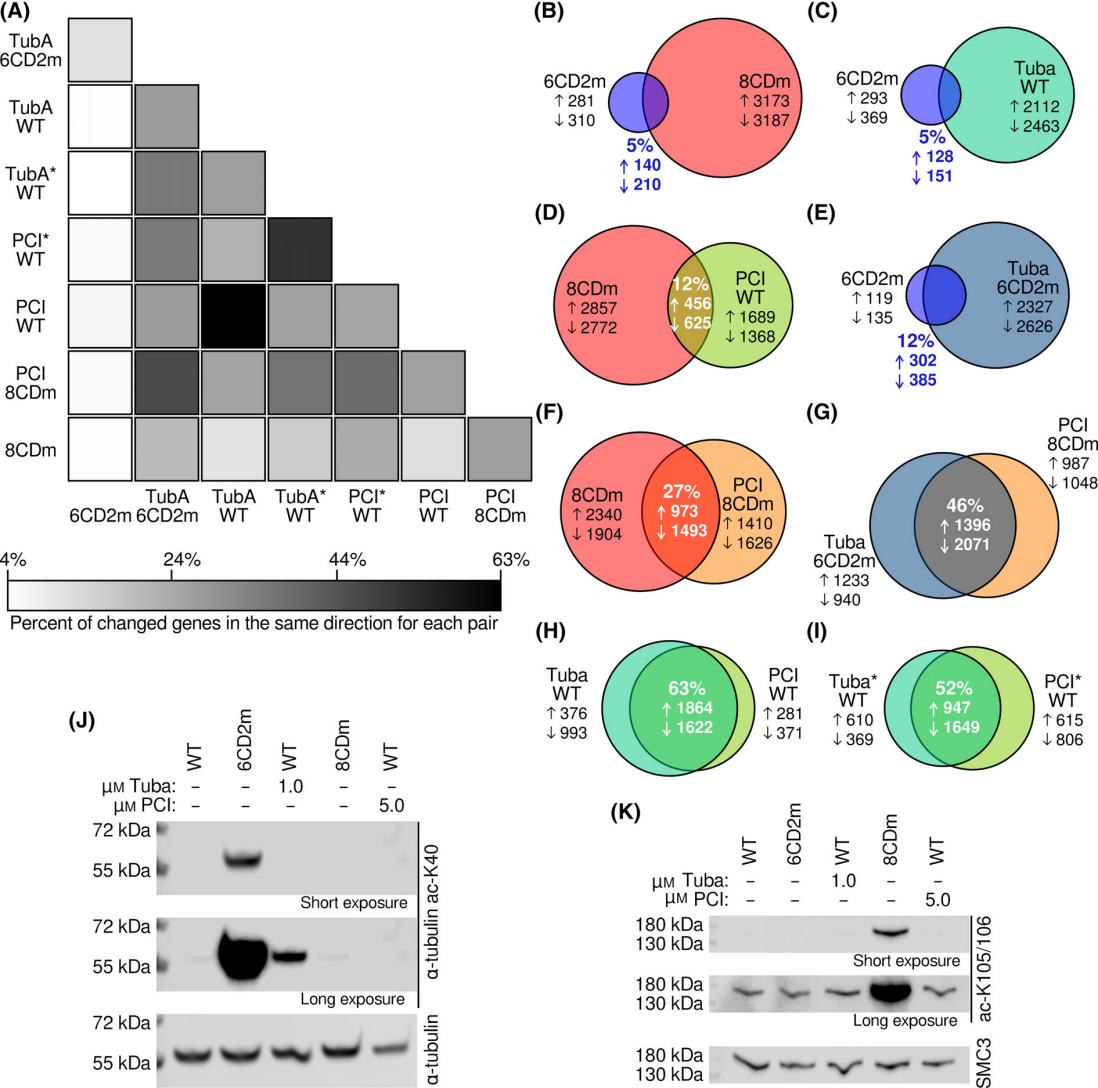
Gene expression analysis reveals off‐target effects of Tubastatin A and PCI‐34051. (A) RNA‐seq was used to identify genes with significant changes in gene expression for each of the conditions listed compared to wild‐type. A heat map of pairwise comparisons is shown where intensity reflects the percent of total changed genes for the pair that are shared in the same direction for both conditions. The scale bar reflects the minimum and maximum percent of shared gene changes in our data set. Where indicated, cells were treated with either Tubastatin A (1.0 μm) or PCI‐34051 (5.0 μm). Treatments were performed for 2 days or 14 days (the latter denoted with *). (B–I) A subset of pairwise comparisons from (A) are represented as Venn diagrams. Circle size is proportional to the total number of gene expression changes compared to untreated WT cells. Overlap area is proportional to the number of expression changes in the same direction at any magnitude that is in common for the two conditions. Arrows represent number of genes up‐regulated or down‐regulated within each portion of the diagrams. The numerical percentage of overlap reflects the sum of up‐regulated and down‐regulated genes. (J) Representative immunoblots of lysates showing levels of α‐tubulin K40 acetylation for each condition, with 14 day treatment of inhibitors. Total α‐tubulin signal served as a loading control. Top and middle panels are different exposure levels of the same blot to allow for comparison of both weak and strong bands. (K) Representative immunoblots of lysates showing levels of SMC3 K105/K106 acetylation for each condition. Total SMC3 signal served as a loading control. Top and middle panels are different exposure levels of the same blot to allow for comparison of both weak and strong bands, with 14 day treatment of inhibitors.

Prior analyses of global gene expression in cells revealed that there was a very different pattern of gene expression changes between genetically inactivated cell lines and treatment of WT cells with KDAC specific inhibitors [14]. To further characterize these differences, we identified gene expression changes, relative to untreated WT cells, in inhibitor‐treated and genetically manipulated HT1080 cells, utilizing a stringent false discovery rate (0.001) (Tables S1–S9). Using these gene lists, we performed pairwise comparisons to determine the percentage of genes that changed expression in the same direction between each pair of treatments (Fig. 2A). As expected and consistent with our previous report (which utilized a less stringent threshold for identifying changed genes), expression changes resulting from genetically inactivating KDAC6 (6CD2m) had little in common with genetically inactivating KDAC8 (8CDm) (Fig. 2B), indicating that these KDACs have distinct, non‐redundant functions. Furthermore, this analysis also highlights the previously observed lack of similarity between 6CD2m and treatment of WT HT1080 with 1.0 μm Tubastatin A, as well as between 8CDm and treatment of WT HT1080 with 5.0 μm PCI‐34051 (Fig. 2C,D), further suggesting that the inhibitors are having consequential off‐target effects in cells even at doses causing little detectable effect on activity of the targeted KDAC. To validate the Tubastatin A result, we treated 6CD2m cells with 1.0 μm Tubastatin A. As there was no active KDAC6 CD2 in these cells and we have previously shown that inactivation of KDAC6 CD1 has far fewer effects than inactivation of CD2, a perfectly selective KDAC6 inhibitor should not have much effect on these cells (although plausibly some non‐catalytic binding could still be disrupted) [14]. Surprisingly, treatment with the low dose of Tubastatin A led to many more changes in gene expression than KDAC6 inactivation alone, and only 12% of the genes which demonstrated expression changes were shared in the same direction between the two conditions (Fig. 2E). Likewise, overlap between untreated 8CDm cells and 8CDm cells treated with the low dose of 5.0 μm PCI‐34051 was only 27% (Fig. 2F). These striking differences of gene expression suggested that, in addition to inhibiting a specific KDAC, the inhibitors must have other consequential effects in cells. In fact, the Tubastatin A‐treated 6CD2m line and the PCI‐34051 treated 8CDm lines had more in common with each other than with the untreated cell lines (Fig. 2G), again demonstrating that the inhibitor treatments were having off‐target effects unrelated to KDAC6 or KDAC8.

Using the same analysis method, we also determined that 63% of gene expression changes were shared between WT cells treated with either Tubastatin A or PCI‐34051 (Fig. 2H), confirming that the effects of the two inhibitors were much more similar to each other than to their respective genetically inactivated cell line, and therefore that the majority of the effect of either molecule has no relationship to the catalytic status of the targeted KDAC. Recognizing that one major difference between the 6CD2m and 8CDm cell lines and inhibitor treatment is chronic vs. acute inactivation, we continuously treated WT cells with the inhibitors for 14 days and then repeated the gene expression analysis. We found that even though some of the changes for the short and long drug treatments (compared to untreated WT) were distinct, PCI‐34051 and Tubastatin A‐treated cells that underwent longer treatment still shared over half of their gene expression changes with each other (Fig. 2A,I). Therefore, the long‐term response of the cells to the two treatments were still more similar to each other than to the genetically inactivated lines. Even after 14 days of inhibitor treatment, the acetylation of α‐tubulin and SMC3 remained much lower than the acetylation in the 6CD2m and 8CDm cell lines, respectively (Fig. 2J,K).

To further characterize differences between genetic inactivation of a KDAC and inhibitor treatment, we considered the gene ontology (GO) biological processes for the genes with expression changes in the same direction for pairs of treatments (Table S10). We first compared the overlap set between 6CD2m and 8CDm, for which only six significant GO terms were identified, primarily clusters of down‐regulated genes associated with mitotic cell division (Fig. S3). No terms were found as significant for the up‐regulated genes in common between the two cell lines. For the cells treated with Tubastatin A or PCI‐34051 for 2 days, 125 significant terms were identified, with up‐regulated genes leading to the majority of identified processes (Fig. S4). All the processes found as significant for both cell lines were also found within the graph for common down‐regulated genes in inhibitor‐treated cells, although the specific GO terms found as significant were slightly different. However, 95% of the significantly impacted biological processes are distinct from the cell line overlap set and include several metabolic, stress response, regulation of post‐translational modification, and DNA repair processes. The large number of biological processes identified suggests that the inhibitors are having common effects on several disparate proteins and/or are both inhibiting a protein involved in regulating numerous processes. After 14 days of inhibitor treatment, the number of significant GO terms identified from common gene expression changes fell by half to 58 terms (Fig. S5). Consistent with the expectation that the cells adapted to the inhibitors in the longer treatment, the stress response, metabolic regulation, and DNA repair processes are notably absent. Most of the significant terms at the long treatment are closely related to other processes identified in the short treatment, but processes in translation and transcription were associated with opposite changes in gene expression. Finally, we examined whether there were any relationships in magnitude of gene expression (as measured by transcripts per million) or the fold change of expression for genes in common between 6CD2m and Tubastatin A treatment or 8CDm and PCI‐34051 treatment, but found no significant trends. Altogether, these results suggest that the two inhibitors have wide‐ranging off‐target effects on cell phenotype that are much greater in number than effects directly associated with inhibition of the target KDAC, at least at the gene expression level.

### Gene expression changes are not primarily due to altered histone acetylation

**Fig. 3:**
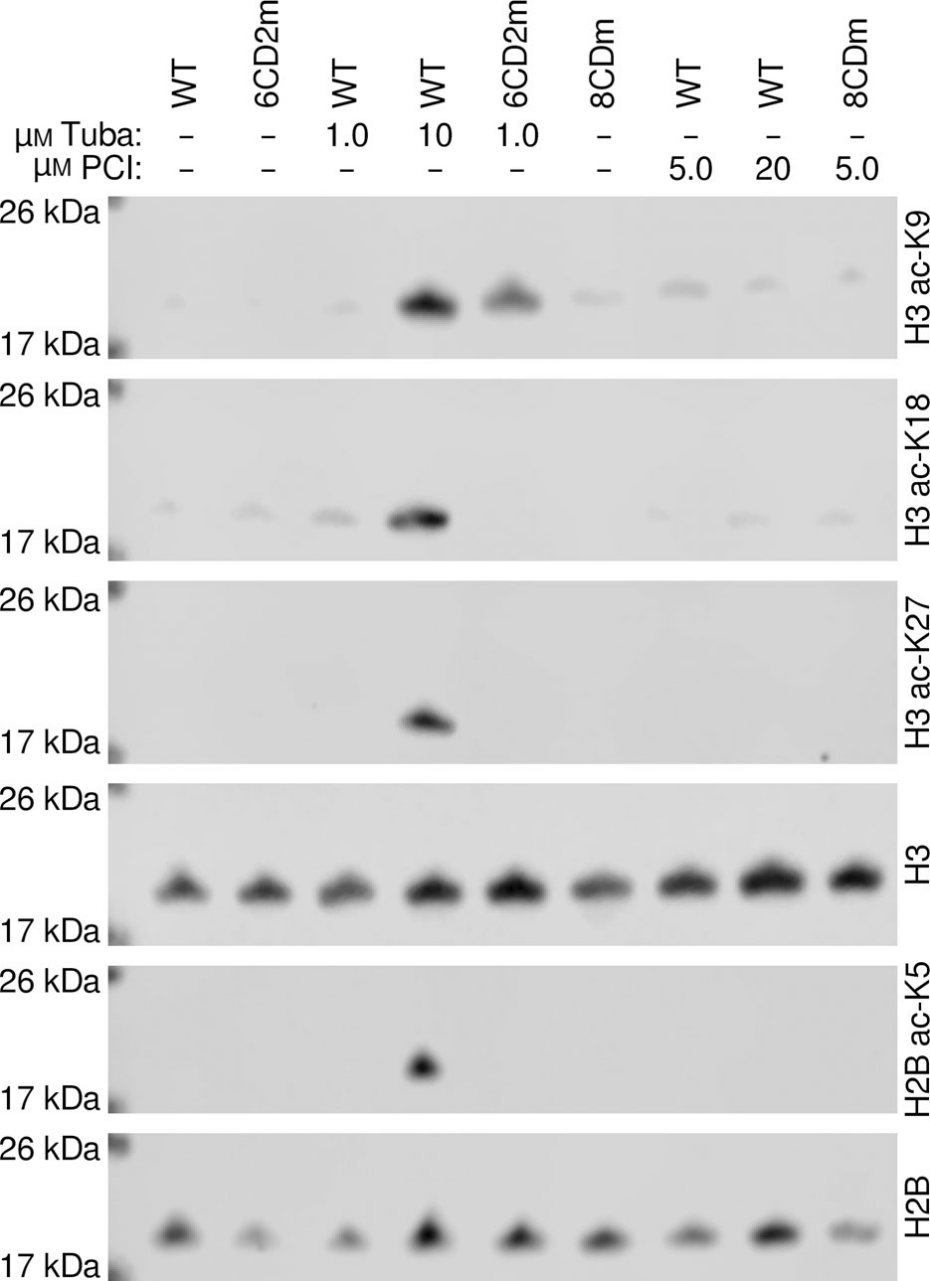
Tubastatin A treatment, but not genetic inactivation of KDAC6 or KDAC8, increases histone acetylation. Cell lines were treated with Tubastatin A (Tuba), PCI‐34051 (PCI), or no inhibitor for 2 days at the specified doses to compare acetylation levels of several known histone acetylation sites. Representative immunoblots of lysates are shown comparing levels of histone acetylated at each indicated position. Total histone levels are shown as loading controls.

One hypothesis for the overall differences in gene expression was that the inhibitors could be inhibiting histone deacetylation, which would in turn affect gene expression. To test this, we immunoblotted lysates from the genetically inactivated cell lines as well as inhibitor‐treated lines to detect changes in histone acetylation (Fig. 3). Importantly, neither the 6CD2m nor the 8CDm cells had increased histone acetylation levels. Treatment with 10 μm Tubastatin A, but not the lower dose, did result in an increase in acetylation at all histone sites that we monitored. We did not see an increase in histone acetylation upon treatment with PCI‐34051, even at a dose of 20 μm.

### Non‐specific binding by KDAC‐selective inhibitors impacts outcomes in therapeutic model systems

**Fig. 4:**
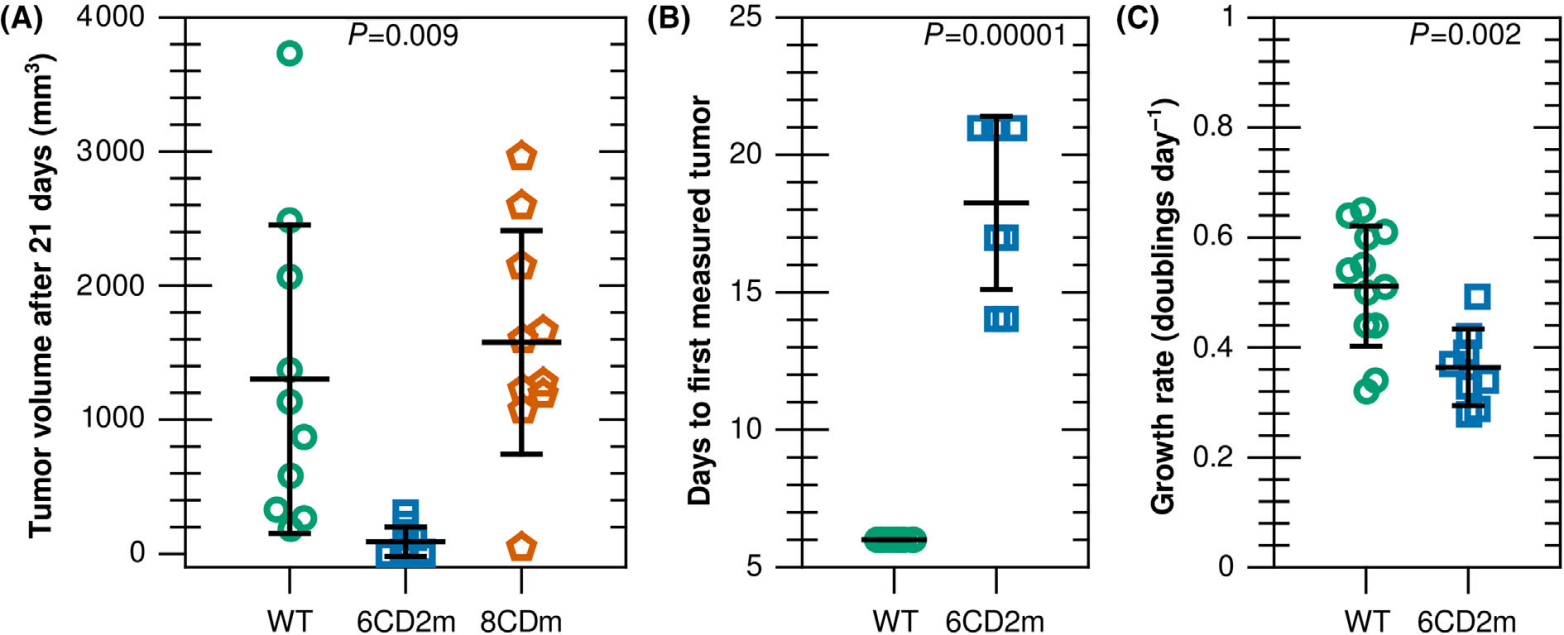
Inactivating KDAC6 in HT1080 cells leads to reduced tumor formation in mouse xenografts. (A) Mice (*n* = 5 per group) were injected bilaterally with WT (green circles), 6CD2m (blue squares), or 8CDm (red pentagons) cells. Tumor volumes were measured 21 days post‐injection and measurements for each tumor are shown. Whiskers represent the mean tumor size and standard deviation. Significant differences are versus WT. (B, C) Mice (*n* = 6 per group) were injected bilaterally with WT or 6CD2m cells. Days to establish tumors and rate of growth are reported for each group. Whiskers represent the mean and standard deviation. Significance was determined by *t*‐test.

After establishing that Tubastatin A and PCI‐34051 lack specificity in cells, we wanted to directly assess the role of KDAC6 and KDAC8 catalysis in cancer cell growth *in vivo*. We injected mice with either 6CD2m or 8CDm cells and compared tumor formation with WT HT1080 cells. HT1080 cells are known to establish large tumors in xenograft studies [29]. The 8CDm line formed tumors that were similar in size and distribution to WT HT1080 cells (Fig. 4). In contrast, the average tumor volume of 6CD2m cells was more than 10‐fold smaller than the WT tumors at the 21‐day endpoint and only an 80% tumor take versus 100% for WT, suggesting the involvement of KDAC6 in tumor establishment and/or tumor growth (Fig. 4, Fig. S7). To resolve these possibilities, we conducted a follow‐up experiment allowing more time for the 6CD2m tumors to establish and grow. In the second experiment, tumor take for WT xenografts was 100%, compared to only 67% with 6CD2m cells. Tumorigenesis was also delayed in KDAC6‐inactive xenografts, as all WT tumors were palpable 6 days after inoculation, while the average time for establishment of 6CD2m tumors was 18 days (Fig. 4B). In addition, once tumors were established the rate of growth was significantly slower in 6CD2m cells (Fig. 4C, Fig. S7). This experiment clearly implicates the catalytic activity of KDAC6 in both tumor formation and tumor growth in this system.

## Discussion

### Off‐target KDAC inhibitor binding leads to phenotypes that are not attributable to KDAC inactivation

In summary, we have compared the biological effects of genetically inactivating KDAC6 or KDAC8 to treatment with putatively specific inhibitors. The two inhibitors are among the least implicated KDAC inhibitors in non‐specific binding in previous studies [11, 12, 13]. Our outcome suggests that the off‐target activity of KDAC inhibitors is even more widespread than previously reported and demonstrates the wide range of cellular changes that occur upon treatment, even when the dose induces little to no measurable expected effect on the acetylation of target proteins. At the protein level, we documented two probable off‐target effects of Tubastatin A. The increase in SMC3 acetylation upon Tubastatin A treatment (Fig. 1A) likely represents an off‐target effect of the KDAC6 inhibitor, possibly by direct inhibition of KDAC8, although we cannot be confident that 10 μm Tubastatin A in the cell culture media results in an intracellular concentration at or above the *in vitro* IC_50_ value for KDAC8 [30]. Our immunofluorescence observations (Fig. 1C) are consistent with previously published data that Tubastatin A treatment increased acetyl‐tubulin primarily in microtubules [31]. The increased intensity of α‐tubulin with the high concentration of Tubastatin A is consistent with reports of microtubule stabilization by the inhibitor, but is likely due to off‐target binding as 6CD2m α‐tubulin is not enhanced relative to WT [31, 32]. The extensive differences in gene expression changes (Fig. 2), and in particular the observation that Tubastatin A and PCI‐34051 cause mostly similar changes to each other but distinct from genetic methods, also implicate off‐target mechanisms as a primary effect of the inhibitors at doses equal to or lower than typically reported in previous research.

In addition to off‐target effects of the inhibitors, several other factors potentially contribute to the differences between genetic inactivation and inhibitor treatment approaches. First, KDAC6 has two catalytic domains, and here we have considered only the second. However, three lines of reasoning suggest that CD1 is not a major factor: (a) the 6CD1m cell line (HT1080 KDAC6H216A) has only 107 genes with significantly altered expression versus 941 genes in the 6CD2m line, and only 70 of those changes are distinct in 6CD1m from 6CD2m (Tables S1 and S9) [14]; (b) there are no reported protein substrates for KDAC6 CD1; and (c) the domain is catalytically competent *in vitro* only with some peptides containing a C‐terminal acetyllysine residue [21, 33]. Therefore, we expect that contributions from CD1 and inhibition of CD1 are negligible in the context of the much greater effects observed from inhibitor treatments, and do not meaningfully impact our conclusions.

A second possibility is off‐target effects in our genetically inactivated cell lines. We established a high degree of confidence of no off‐target effects during CRISPR/Cas9 editing in our original report of the cell lines [14]. Although some random mutation is inevitable for cell line populations separated by up to 3 months, as is the case here, any particular mutation is present in only a subset of cells, and so is unlikely to greatly influence population‐level metrics such as those reported here. Strong support for the lack of any unexpected genetic effect as a driver of our results comes from comparisons in Fig. 2. Specifically, 6CD2m and 8CDm cells have little overlap in gene expression changes compared to Tubastatin A or PCI‐34051 treated WT cells, respectively, but upon treatment with inhibitor have much greater similarity (Fig. 2A). Moreover, 6CD2m and 8CDm have few similar gene expression changes (Fig. 2B), whereas Tubastatin A‐treated 6CD2m has nearly 50% similarity to PCI‐34051‐treated 8CDm (Fig. 2G). These results indicate that while there may be minor contributions from off‐target or unexpected genetic mutations, the common off‐target effects of the inhibitors are much greater.

Third, the genetically modified cells may have adapted to or compensated for the loss of KDAC function, and certainly, some amount of adaption is unavoidable. Such adaptation could serve to minimize the number of gene expression changes, resulting in an artificially large difference when comparing to inhibitor treatment. However, the fact that most gene expression changes in cells treated with each inhibitor were much more similar to each other than to the cell lines (Fig. 2), even at the dosing of inhibitor that caused little or no detectable change in acetylation on known KDAC targets (Fig. 1), justifies the conclusion that the two inhibitors are primarily having off‐target effects.

### Neither KDAC6 nor KDAC8 deacetylates histones in HT1080 cells

Prior work has suggested that HDAC6 and HDAC8 each directly deacetylate histones in cells [4, 34, 35, 36, 37, 38]. However, other studies have reported that HDAC6 knockout cell lines do not exhibit altered histone acetylation and that HDAC6 is predominantly located in the cytoplasm [39, 40, 41]. Although KDAC8 has at least been observed as localized substantially to the nucleus in addition to the cytoplasm, the evidence for histone deacetylation by KDAC8 is almost entirely from *in vitro* experiments [4, 38, 42, 43, 44, 45]. Our result of unaltered histone acetylation in either the 6CD2m or 8CDm cell lines (Fig. 3) is direct evidence that neither KDAC6 nor KDAC8 is responsible for histone deacetylation in HT1080 cells, and is consistent with the reported knockout studies for KDAC6. Our observation of increased histone acetylation with Tubastatin A treatment (Fig. 3) is consistent with previous reports that moderate to high doses of Tubastatin A led to increased histone acetylation at several sites [12, 19, 36]. Thus, Tubastatin A is most likely affecting histone acetylation levels by inhibiting another KDAC. Previous findings that Tubastatin A did not bind KDACs known to be responsible for histone deacetylation in cells were based on a 1.0 μm dose [11]. The off‐target effects are dose‐dependent, and our results emphasize how critical dosing is for performing and interpreting inhibitor‐based experiments in cells. Our observed lack of enhanced histone acetylation with PCI‐34051 treatment is also consistent with a previous report, where an increase in histone acetylation was only detected when a ≥50 μm dose of PCI‐34051 was used [20]. In aggregate, we conclude that neither KDAC6 nor KDAC8 deacetylates histones, and that changes in histone acetylation can be used as a crude screening tool to identify non‐selective binding of any putatively selective inhibitor targeting KDAC6 or KDAC8. Moreover, conclusions regarding the targets or functions of KDAC6 and KDAC8 drawn from any study in which histone acetylation has been altered by inhibitor treatment may need to be treated as suspect.

### The primary off‐target effects of the inhibitors are KDAC‐independent

Together, the gene expression data strongly suggested that these inhibitors may have a common target or set of targets besides KDAC6 and KDAC8 in cells. While the increase in histone acetylation upon treatment with 10 μm Tubastatin A would likely result in some gene expression changes, this cannot explain all of the gene expression changes that we observed, as cells used in the gene expression experiments were treated with the lower dose of the inhibitor that does not appear to impact histone acetylation. Furthermore, the high overlap in gene changes between the two inhibitors (Fig. 2), coupled with the fact that histone acetylation levels did not change upon treatment with PCI‐34051 (Fig. 3), suggests that these inhibitors are primarily having additional, partially redundant, off‐target effects via binding to proteins outside of the KDAC family. The extensive differences in significant GO terms identified from the common gene expression changes from treatment by either inhibitor (Figs S4, S5), compared to common effects of genetic inactivation of either KDAC (Fig. S3), further indicates that the majority of cellular effects are not related to inhibition of the target KDAC.

### Off‐target binding and incomplete inhibition produce misleading outcomes

The 8CDm xenograft result (Fig. 4A) is in contrast to previous reports of xenografts of neuroblastoma cells forming significantly smaller tumors when cells were pretreated with RNAi against KDAC8 or when mice were treated with PCI‐48012, a structurally similar compound to PCI‐34051 which, unlike PCI‐34051, is sufficiently stable for *in vivo* use [46]. While future work will be required to determine whether these results are cell‐type specific, the neuroblastoma results may not be attributable to the catalytic function of KDAC8. Our result implies that a therapeutic strategy for KDAC8 which leads to degradation of the enzyme would be more effective than treatment with a competitive inhibitor.

The KDAC6 xenograft results (Fig. 4) also allowed us to resolve prior conflicting reports in the literature concerning the role of KDAC6 in tumorigenesis. Several previously reported mouse xenograft studies using different cancer cell types reported only very small decreases in tumor size upon Tubastatin A treatment alone, even using high treatment doses, consistent with our observations that Tubastatin A incompletely inhibits KDAC6 and that even complete KDAC6 inhibition only reduces the tumor growth rate to approximately 75% [47, 48, 49]. However, two prior observations suggest a role for KDAC6 in tumorigenesis. First, cells in which KDAC6 was knocked down formed smaller tumors than untreated counterparts [50]. Second, KDAC6‐null mice formed significantly less carcinogen‐induced skin tumors than WT mice, but tumor volume appeared to increase at a similar rate in both populations once tumors were established [50]. This is consistent with our observation that tumor formation is more strongly impaired than growth (after tumors are established) when KDAC6 is inactivated. Inhibitor studies were not able to reveal the defect in tumor formation.

### Implications for the design of KDAC inhibitors

The large majority of competitive KDAC inhibitors that have been developed, including Tubastatin A and PCI‐34051, rely on a zinc‐binding group to chelate the metal in the active site of the enzyme [10]. Recent literature suggests that this mechanism of action may lead to off‐target binding in cells, as the zinc‐binding group could bind to other metal‐containing proteins in cells and/or chelate free metals, which at high enough concentrations could be sufficient to affect cell function by changing the intracellular concentration of essential metal cofactors [11, 13]. Our results demonstrate meaningful and severe cellular consequences occur as a result of off‐target binding, demonstrating that the off‐target binding effects cannot be ignored when interpreting the results of KDAC inhibition studies. Our results highlight the need for a different type of selective KDAC inhibitor, that does not rely on the zinc‐binding group, to alleviate most of the non‐specific effects of KDAC inhibitors and truly target single KDACs *in vivo*. It is probable that off‐target effects are also responsible for most of the effects of pan‐KDAC inhibitors, including approved drugs, as most inhibitors share molecular features likely driving off‐target binding and evidence of primarily off‐target effects has been reported for KDAC drug candidates [17].

Although Tubastatin A and PCI‐34051 have generally appeared to be among the most selective of commonly used KDAC inhibitors, we confidently conclude that the molecules are not selectively inhibiting KDAC6 and KDAC8, as cells treated with these inhibitors did not mimic the cell lines in any of the ways that we queried them, while simultaneously demonstrating significant overlap in effect with treatment by the other inhibitor and incomplete inhibition of the target KDAC. It is clear that a comparison of *in vitro* IC_50_ values within the KDAC family is insufficient to establish selectivity, that IC_50_ values established *in vitro* do not translate proportionally to *in vivo* selectivity, and that KDAC inhibitors should be evaluated for binding to proteins outside the KDAC family. We suggest that all KDAC inhibitors should be characterized through at least one of either a comparison to the genetic inactivation, such as we have described here, or whole‐cell binding screen such as reported elsewhere [11].

The biological effects presented here, as well as our previously reported gene ontology analysis demonstrating distinct cellular functions associated with KDAC inhibitors separate from the KDAC catalytic activity, underscore the importance of both understanding the catalytic roles of individual KDACs and effectively targeting them for therapy [14]. The ability to directly compare turning off a single KDAC in cells to using a purported selective inhibitor allows for information not obtainable with previous methods that relied on lowering or eliminating expression of the entire protein in cells (i.e. knockdown/knockout), which eliminates both catalytic and non‐catalytic functions of the enzyme. These cell lines revealed the true cellular consequence of turning off a single KDAC while retaining its expression, and exposed a critical lack of selectivity of KDAC inhibitors that justifies re‐evaluation of studies relying on those inhibitors for biological and therapeutic effects.

## Conflict of interest

The authors declare no conflict of interest.

## Author contributions

TBT and TJW conceived the project. BCM, MEB, TBT, and TJW designed and supervised the experiments. ECM, KEB, MAM, MCB, TBT, and TMH performed the experiments. KEB, MAM, MF, TBT, TJW, TMH, and VHB analyzed the data. KEB wrote the initial draft. All authors reviewed and revised the manuscript.

## Supporting information


**Fig. S1.** Uncropped immunoblots for data shown in Fig. 1.
**Fig. S2.** Uncropped immunoblots for data shown in Fig. 2.
**Fig. S3.** GO graph for genes with expression changes in the same direction in 6CD2m and 8CDm.
**Fig. S4.** GO graph for genes with expression changes in the same direction in wild‐type HT1080 treated with 1.0 μm Tubastatin A or 5.0 μm PCI‐34051 for 2 days.
**Fig. S5.** GO graph for genes with expression changes in the same direction in wild‐type HT1080 treated with 1.0 μm Tubastatin A or 5.0 μm PCI‐34051 for 14 days.
**Fig. S6.** Uncropped immunoblots for data shown in Fig. 3.
**Fig. S7.** Growth curves of individual tumors.

**Table S1.** Significant gene expression changes in 6CD2m relative to wild‐type.
**Table S2.** Significant gene expression changes in 8CDm relative to wild‐type.
**Table S3.** Significant gene expression changes in 6CD2m treated with 1.0 μm Tubastatin A for 2 days relative to untreated wild‐type.
**Table S4.** Significant gene expression changes in 8CDm treated with 5.0 μm PCI‐34051 for 2 days relative to untreated wild‐type.
**Table S5.** Significant gene expression changes in wild‐type HT1080 treated with 1.0 μm Tubastatin A for 2 days relative to untreated wild‐type.
**Table S6.** Significant gene expression changes in wild‐type HT1080 treated with 5.0 μm PCI‐34051 for 2 days relative to untreated wild‐type.
**Table S7.** Significant gene expression changes in wild‐type HT1080 treated with 1.0 μm Tubastatin A for 14 days relative to untreated wild‐type.
**Table S8.** Significant gene expression changes in wild‐type HT1080 treated with 5.0 μm PCI‐34051 for 14 days relative to untreated wild‐type.
**Table S9.** Significant gene expression changes in 6CD1m relative to wild‐type.
**Table S10.** Significant GO terms of common gene expression changes.

## Data Availability

The data that support the findings of this study are openly available in Gene Expression Omnibus at www.ncbi.nlm.nih.gov/geo/, reference numbers GSE228549 and GSE251743, and in Open Science Framework at 10.17605/OSF.IO/Y6DSA, reference number Y6DSA.
