# Supplementary material for: Lysine deacetylase inhibitors have low selectivity in cells and exhibit predominantly off‐target effects
Source: FEBS Open Bio. 2024 Oct 31;15(1):94–107. doi: 10.1002/2211-5463.13896 (PMC11705486; doi:10.1002/2211-5463.13896)
Supplement: Supplementary file 1 — Fig. S1. Uncropped immunoblots for data shown in Fig. 1. Fig. S2. Uncropped immunoblots for data shown in Fig. 2. Fig. S3. GO graph for genes with expression changes in the same direction in 6CD2m and 8CDm. Fig. S4. GO graph for genes with expression changes in the same direction in wild‐type HT1080 treated with 1.0 μm Tubastatin A or 5.0 μm PCI‐34051 for 2 days. Fig. S5. GO graph for genes with expression changes in the same direction in wild‐type HT1080 treated with 1.0 μm Tubastatin A or 5.0 μm PCI‐34051 for 14 days. Fig. S6. Uncropped immunoblots for data shown in Fig. 3. Fig. S7. Growth curves of individual tumors. [file FEB4-15-94-s001.pdf]

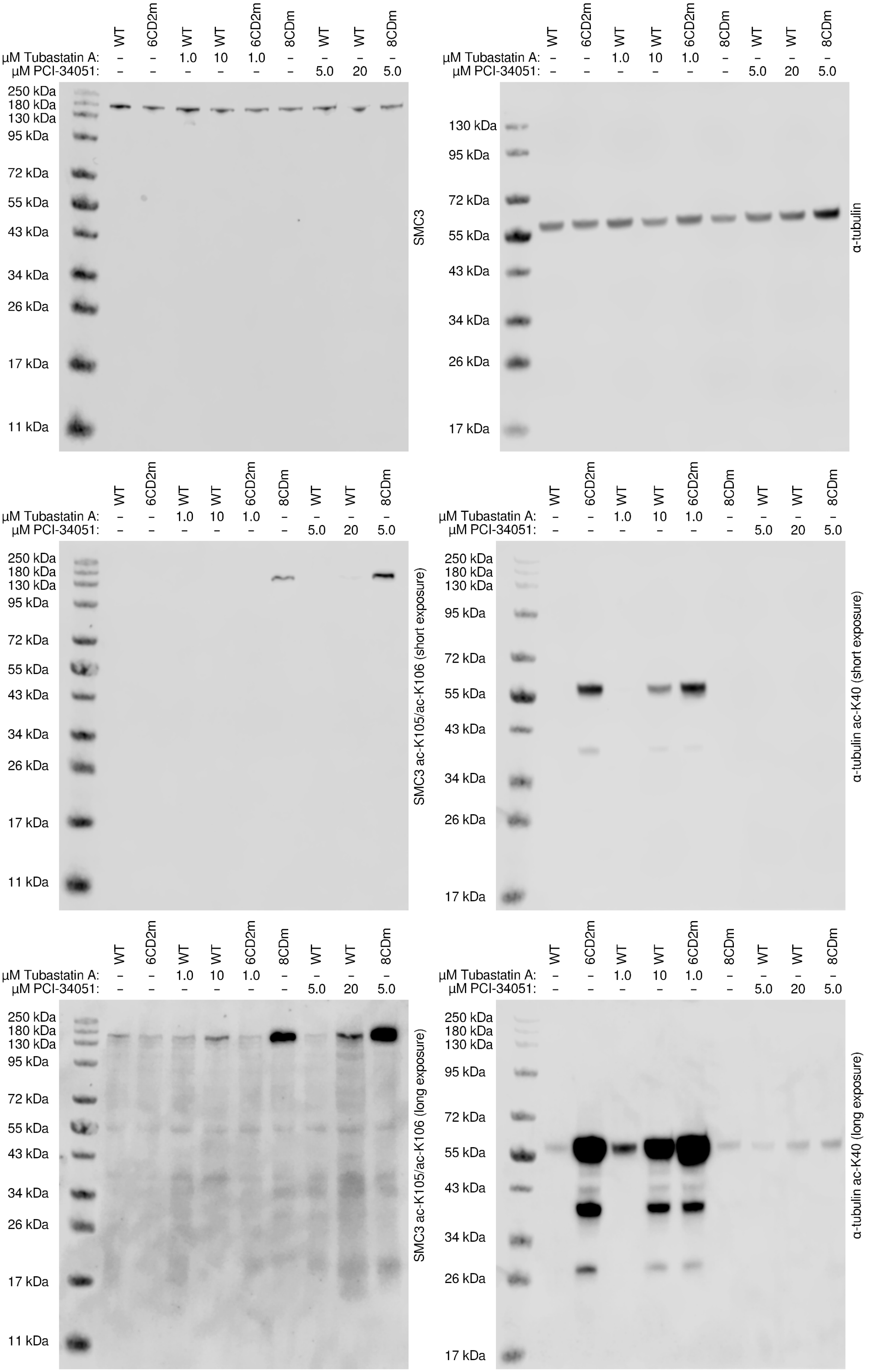

Figure S1. Uncropped immunoblots for data shown in Figure 1. α-tubulin is 50 kDa but runs higher, and SMC is 142 kDa.

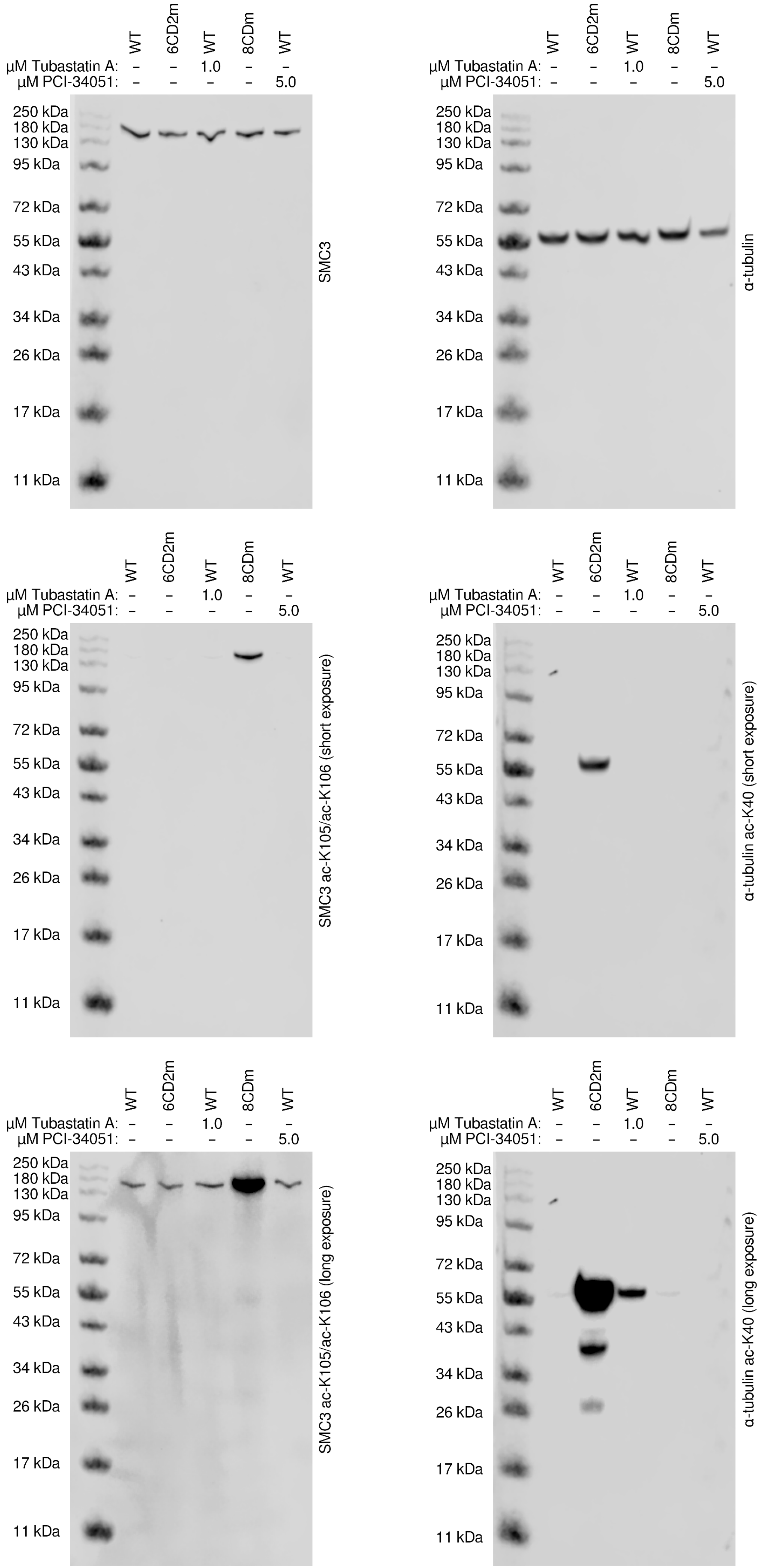

Figure S2. Uncropped immunoblots for data shown in Figure 2. α-tubulin is 50 kDa but runs higher, and SMC is 142 kDa.

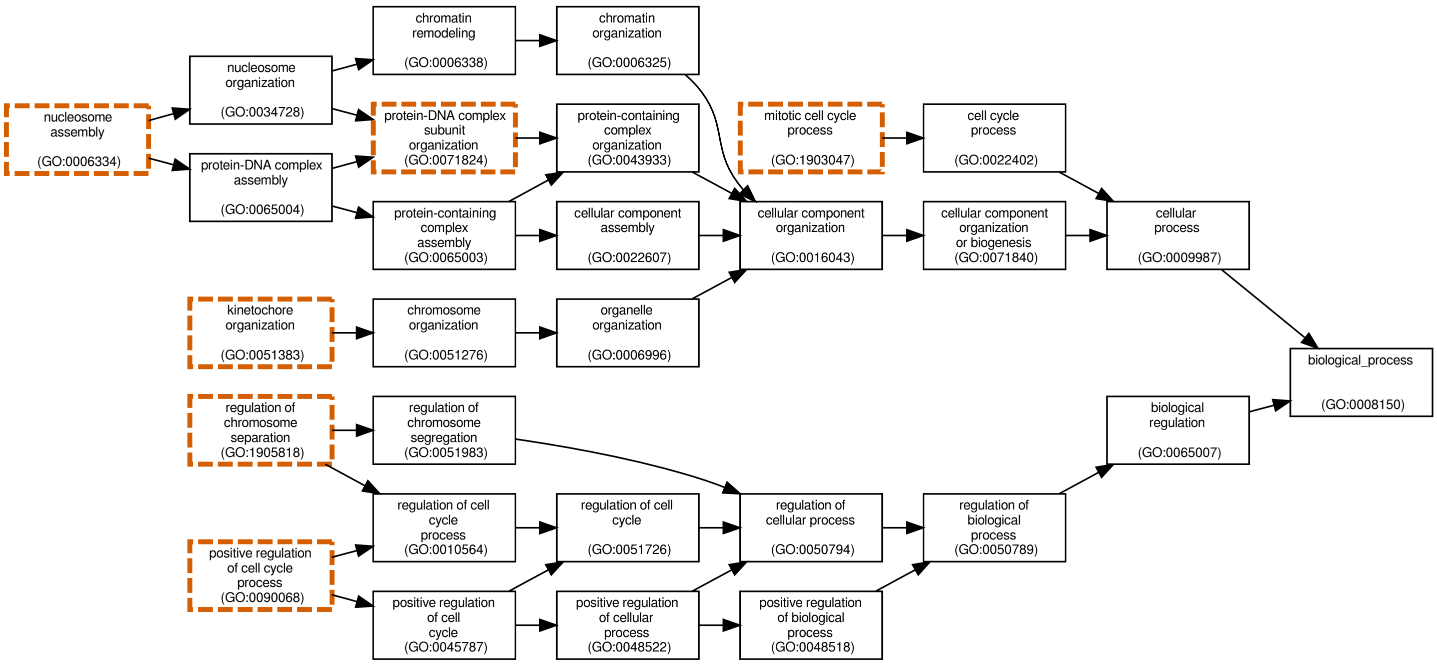

Figure S3. GO graph for genes with expression changes in the same direction in 6CD2m and 8CDm. Red dashed boxes are terms that were significant for down-regulated genes. Black boxes are terms that were not significant according to the method used, but which exist along the shortest path between a significant term and biological_process. Lines represent is_a and part_of relationships. This figure is text-searchable.

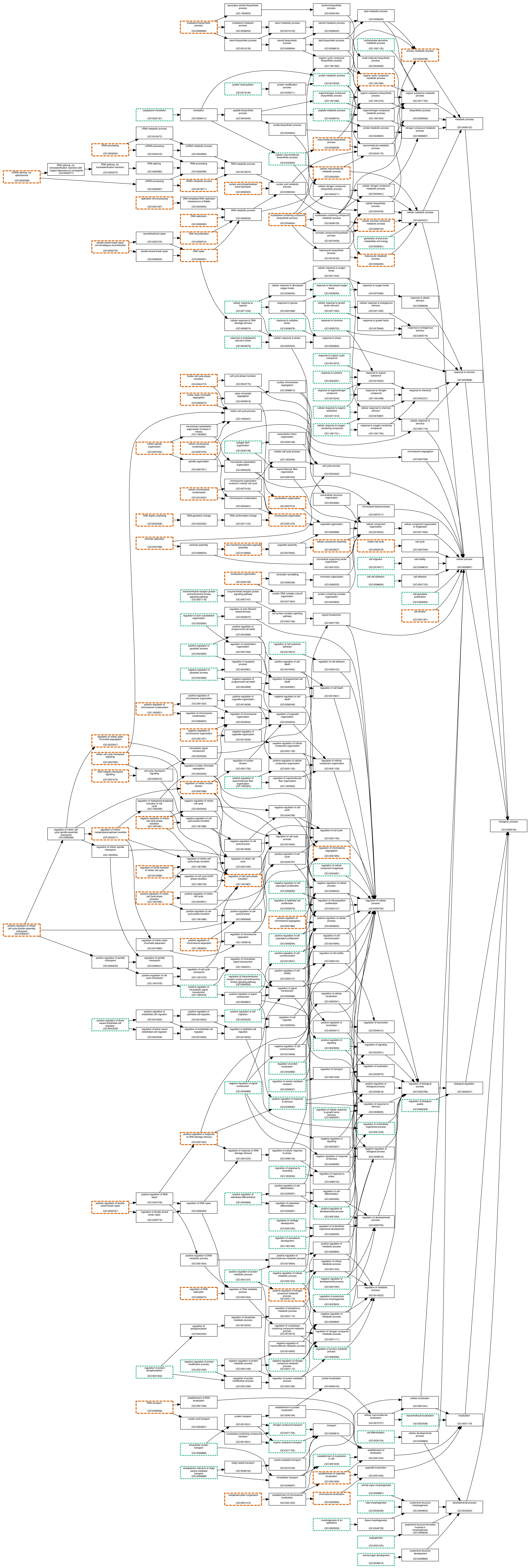

Figure S4. GO graph for genes with expression changes in the same direction in wild-type HT1080 treated with 1.0 μM Tubastatin A or 5.0 μM PCI-34051 for 2 days. Red dashed boxes are terms that were significant for down-regulated genes. Dotted green boxes are terms that were significant for up-regulated genes. Black boxes are terms that were not significant according to the method used, but which exist along the shortest path between a significant term and biological_process. Lines represent is_a and part_of relationships. This figure is text-searchable.

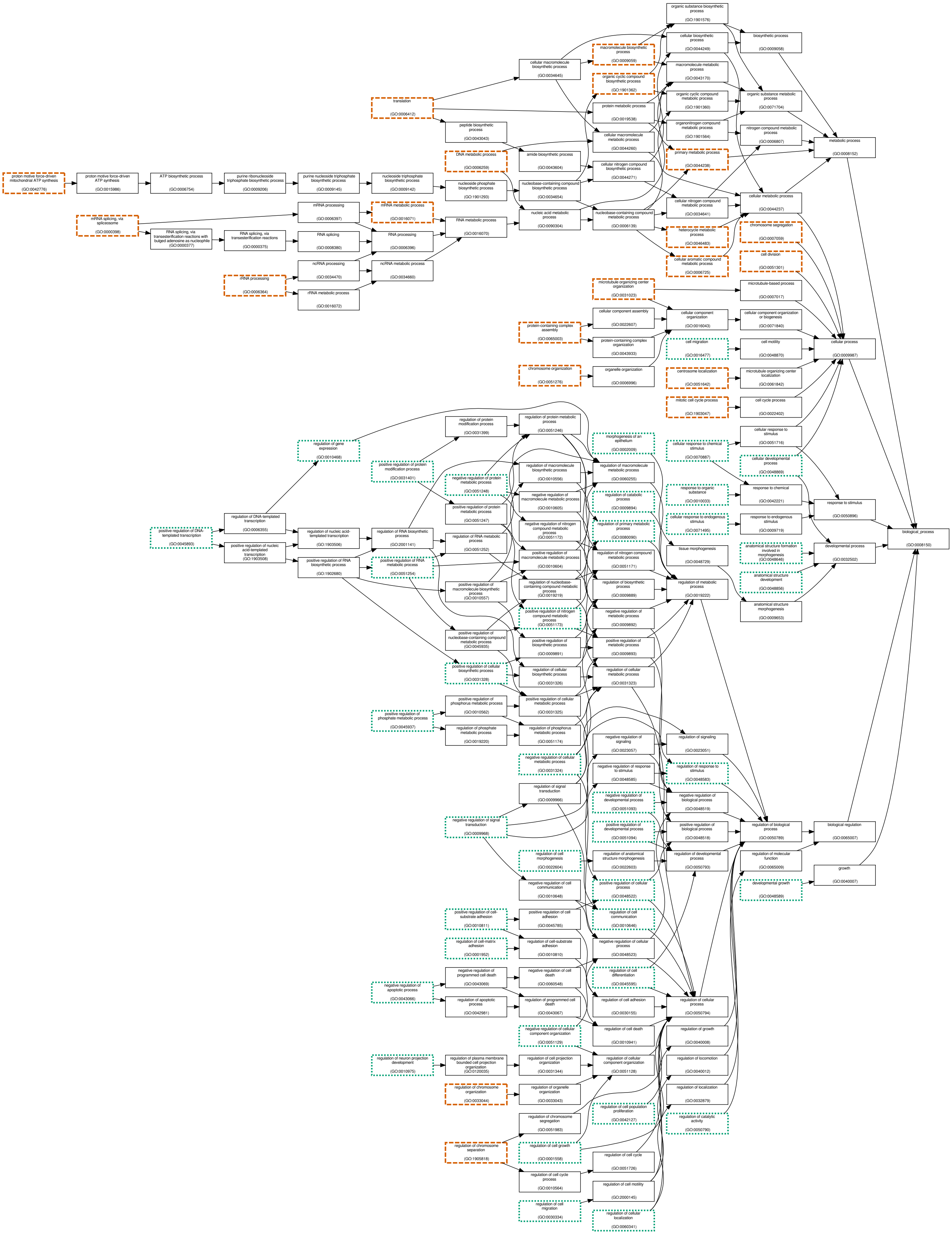

Figure S5. GO graph for genes with expression changes in the same direction in wild-type HT1080 treated with 1.0 µM Tubastatin A or 5.0 µM PCI-34051 for 14 days. Red dashed boxes are terms that were significant for down-regulated genes. Dotted green boxes are terms that were significant for up-regulated genes. Black boxes are terms that were not significant according to the method used, but which exist along the shortest path between a significant term and biological_process. Lines represent is_a and part_of relationships. This figure is text-searchable.

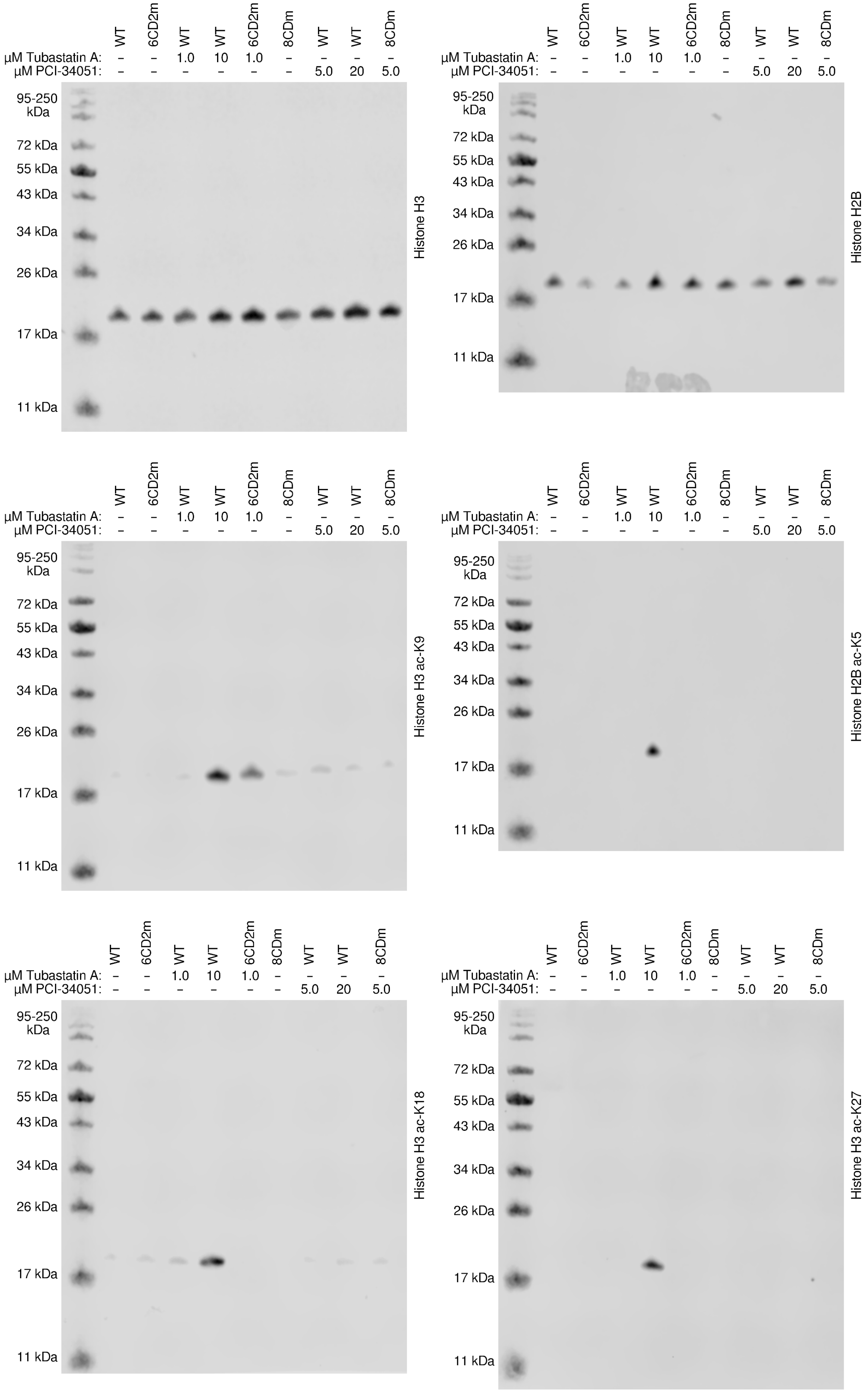

Figure S6. Uncropped immunoblots for data shown in Figure 3. Each histone protein is ~15 kDa but runs higher.

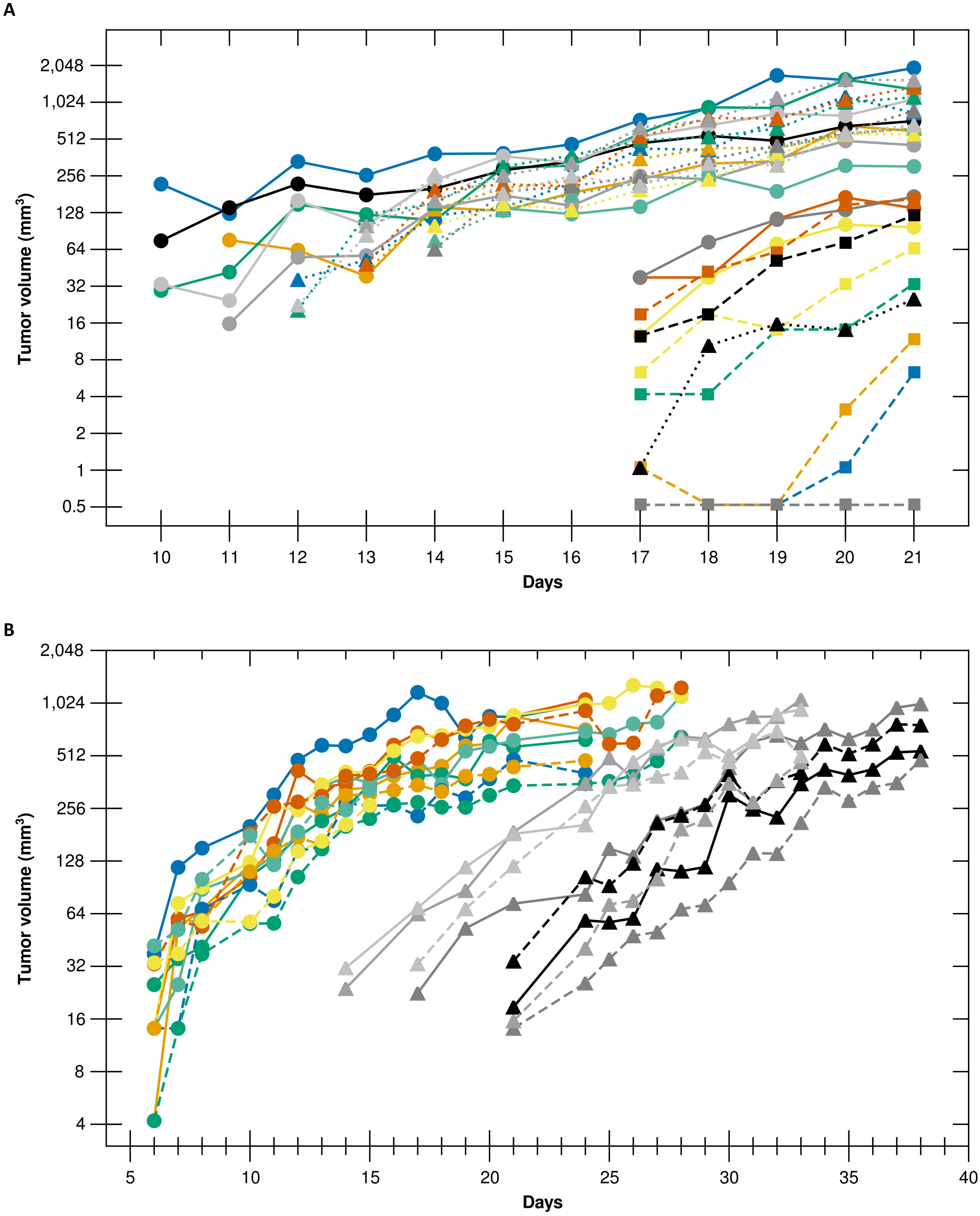

Figure S7. Growth curves of individual tumors. (A) WT (circles, solid lines), 6CD2m (rectangles, dashed lines), and 8CDm (triangles, dotted lines) in first xenograft experiment. (B) WT (circles, in colors) and 6CD2m (triangles, in grays) in second xenograft experiment.
